# CIRBP Knockdown Attenuates Tumourigenesis and Improves the Chemosensitivity of Pancreatic Cancer via the Downregulation of DYRK1B

**DOI:** 10.3389/fcell.2021.667551

**Published:** 2021-08-20

**Authors:** Xiang Chen, Hongyu Xie, Xin Wang, Zhinan Zheng, Sanqing Jin

**Affiliations:** Department of Anesthesia, The Sixth Affiliated Hospital, Sun Yat-sen University, Guangzhou, China

**Keywords:** cold-inducible RNA binding protein, pancreatic ductal adenocarcinoma, dormancy, tumorigenesis, chemoresistance

## Abstract

Pancreatic ductal adenocarcinoma (PDAC) is one of the most lethal malignancies worldwide with very limited treatment options. Cold-inducible RNA binding protein (CIRBP) plays promoting roles in several types of cancers, but its function remains unclear in PDAC. Here, we found that the expression of CIRBP was upregulated in PDAC tumor tissues and was significantly associated with poor prognosis. Knockdown of CIRBP in PANC-1 and SW1990 cells inhibited proliferation, migration and invasion *in vitro* and suppressed tumor growth *in vivo*. Moreover, CIRBP knockdown enhanced the antitumour effects of gemcitabine treatment in PANC-1 and SW1990 cells, whereas CIRBP overexpression exerted the opposite effects. Mechanistically, CIRBP promoted PDAC malignancy and chemoresistance via upregulation of dual-specificity tyrosine-Y-phosphorylation regulated kinase 1B (DYRK1B). Indeed, knockdown of CIRBP sensitized pancreatic tumors to gemcitabine treatment by diminishing DYRK1B expression and increasing the ratio of ERK/p38 activity. Our findings suggest that CIRBP overexpression facilitates PDAC progression and gemcitabine resistance by upregulating DYRK1B expression and inhibiting the ERK/p38 signaling pathway, highlighting CIRBP as a potential new therapeutic target for PDAC.

## Introduction

Pancreatic ductal adenocarcinoma (PDAC) is one of the most lethal malignancies and its 5-year survival rate is approximately 7–9% (Bray et al., 2018; Siegel et al., 2020). Because of the insidious onset and early local infiltration of PDAC, the diagnosis of most PDAC patients is delayed, and the opportunity for radical surgery is lost (Conroy et al., 2011; Von Hoff et al., 2013; Neoptolemos et al., 2017). Although significant improvements in new treatment modalities have been achieved (Hidalgo, 2010; Ryan et al., 2014), the low response rate to chemotherapy due to the development of gemcitabine (Gem) resistance is common in the clinic, limiting therapeutic efficacy (de Sousa and Monteiro, 2014). The progression of PDAC involves a complex regulatory network consisting of many signaling pathways (Spivak-Kroizman et al., 2013). Therefore, it is of vital importance to explore the molecular mechanisms of carcinogenesis and chemosensitivity in PDAC to discover novel and more effective therapeutic approaches.

Cold-inducible RNA binding protein (CIRBP), also called DNA damage-induced transcript (A18 hnRNP), is a nuclear protein, but it tends to translocate to the cytoplasm in response to cellular stresses, such as cold, ultraviolet radiation, and hypoxia (Chappell et al., 2001; Yang and Carrier, 2001; Lujan et al., 2018). The role of CIRBP in carcinogenesis appears to be mainly driven by its functions as an RNA binding proteins (RBPs), promoting the stability and translation of specific mRNAs encoding cancer-associated proteins. Notably, CIRBP was originally considered as a tumor suppressor that inhibits tumor cell proliferation by prolonging the G1 phase of the cell cycle and participating in the DNA damage response (Yang et al., 2006). However, recent studies found that CIRBP was overexpressed in prostate cancer (Zeng et al., 2009), liver cancer (Lee et al., 2015), breast cancer (Guo et al., 2010), and bladder cancer (Lu et al., 2018) and was involved in upregulating telomerase activity, promoting proliferation, inhibiting apoptosis, and promoting epithelial-mesenchymal transition (EMT) (Zhang et al., 2016). Thus, high CIRBP expression in these tumors results in low chemosensitivity and poor prognosis (Zeng et al., 2009; Zhu et al., 2016; Zhou et al., 2018).

The highly aggressive nature of pancreatic cancer is due to not only its deep anatomical location, but also the large number of quiescent cancer cells (Stanton et al., 2003; Ryan et al., 2014). Pancreatic cancer cells can enter quiescence, a process of entry into reversible G0 cell cycle arrest, upon hypoxia exposure or nutritional deprivation and thus become chemoresistant, a property that also contributes to tumor recurrence when these cells re-enter the cell cycle (Ewton et al., 2011; Rankin and Giaccia, 2016; Recasens and Munoz, 2019). The protein kinase dual-specificity tyrosine-Y-phosphorylation regulated kinase 1B (DYRK1B) plays a key role in the G0/G1–S phase transition, which is necessary to maintain cancer cells in a quiescent state (Becker, 2018). Phosphorylation by DYRK1B results in the degradation of cyclin D1 and stabilization of p27, leading to G0 arrest, thereby reducing chemosensitivity in many cancers especially in pancreatic cancer (Deng et al., 2009, 2014; Becker, 2012, 2018; Ashford et al., 2014).

In this study, we explored the roles of CIRBP in PDAC tumourigenesis and chemosensitivity *in vivo* and *in vitro*. Given the induction of cancer cell dormancy by DYRK1B in various cancer types (Becker, 2018), we also investigated the interactions between CIRBP and DYRK1B as well as the resulting impacts of these interactions on the tumourigenesis and chemoresistance of PDAC cells.

## Materials and Methods

### Human Tissue Specimens and Immunohistochemistry (IHC) Analysis

Pancreatic cancer tissue microarrays (TMAs) were obtained from Shanghai Outdo Biotech Company. The TMAs contained 90 primary pancreatic cancer and 60 normal adjacent pancreatic tissue specimens. Clinicopathological data and survival data of 90 patients were provided by the manufacturer. Overall survival (OS) was defined as the interval from the date of surgery to death. IHC staining of these specimens was performed with primary antibodies against CIRBP (catalog no. 10209-2-AP; Proteintech Group, United States), and the images were obtained under a microscope using CellSens Dimension software (Olympus, Hamburg, Germany). The IHC scoring was based on the intensity and extent of staining according to the Fromowitz standard (Williams et al., 1988). Staining intensity was graded as follows: 0, negative staining; 1, weak staining; 2, moderate staining; and 3, strong staining. The proportion of positive cells in each per specimen was evaluated semi-quantitatively as follows: 0, <1%; 1, 1–25%; 2, 26–50%; 3, 51–75%; and 4, >75%. The histological score (H-score) for each specimen was calculated by multiplying the staining intensity score and the positive staining proportion score. The samples were further defined by the *H*-score as negative (−, score: 0), weak (+, score: 1–4), moderate (++, score: 5–8), and strong (+++, score: 9–12) and divided by the H-score into the CIRBP-high group (H-score ≥ 3) and CIRBP-low group (H-score < 3).

### Cell Lines and Cell Culture

The human pancreatic cancer cell lines PANC-1, SW1990 and AsPC-1 were purchased from the Stem Cell Bank, Chinese Academy of Sciences in Shanghai, China. MIA PaCa-2 cells were provided by Dr. Ruihua Xu (Ju et al., 2016) (Sun Yat-sen University Cancer Center, Guangzhou, China). The Capan-2 cells and the immortalized human pancreatic duct epithelial cell line (HPDE6-C7) were generous gifts from Dr. Rufu Chen (Wei et al., 2018). All cell lines were grown in Dulbecco’s modified Eagle’s medium (DMEM) (Gibco, China) supplemented with 10% heat-inactivated fetal bovine serum (FBS) (Gibco, South America) and 1% penicillin/streptomycin (Gibco, United States), incubated in a humidified incubator at 37°C with a mixture of 95% air and 5% carbon dioxide (Forma Scientific/Thermo Scientific, Waltham, MA, United States), and harvested by 0.25% trypsin-EDTA (ethylenediaminetetraacetic acid) (Gibco, China). All cells were stored in a liquid nitrogen container.

### Cell Transfections, and Selection of Stable Cell Lines

Lentiviral human CIRBP-targeting short hairpin RNA (shRNA) was purchased from GeneChem (Shanghai, China). Double-stranded DNA oligos carrying interference sequences were synthesized to obtain CIRBP knockdown. The target shRNAs against the human CIRBP gene (GenBank accession NM_001300829) for RNAi were CIRBP sh#1: 5′-CATGA ATGGGAAGTCTGTA-3′ and CIRBP sh#2: 5′-TCTCAAAGTA CGGACAGAT-3′. The sequence 5′-TTCTCCGAACGTGTCAC GT-3′, which had no significant homology to any known human or mouse genes, was used as a negative control. To overexpress CIRBP or DYRK1B, the cDNA of a fragment encoding the full-length human CIRBP or DYRK1B open reading frame (ORF) sequence was amplified by PCR and then cloned into a GV341 lentiviral expression vector (Shanghai Genechem) to obtain recombinant lentivirus. According to the expression levels of CIRBP in several PDAC cell lines, we selected two cell lines, PANC-1 and SW1990, to construct stable CIRBP overexpression or knockdown cell lines to exclude the influence of endogenous CIRBP expression on the recombinant lentiviral transfection effect, which PANC-1 showed the lowest expression levels of CIRBP and SW1990 showed the highest. At 72 h post-infection, the cells were treated with 2 μg/ml puromycin for 7 days to obtain stable cell lines. Quantitative real-time PCR (RT-qPCR) and western blotting were used to verify the expression levels of CIRBP and DYRK1B. The stable cell lines were stored in a liquid nitrogen container.

### Colony Formation Assay

To evaluate the colony-forming ability, the transfected cells were plated onto 6-well plates at a concentration of 500 cells per well in triplicate, and then incubated at 37°C for 14 days to form colonies. The colonies were then washed twice with phosphate buffered saline (PBS) and stained with crystal violet staining solution (0.1% w/v, Beyotime Biotechnology, China). The number of colonies containing at least 50 cells was counted under a microscope. All of the experiments were repeated three times.

### Cell Migration and Invasion Assay

For the scratch wound assay, the transfected cells were seeded (2 × 10^5^ cells per well) in 24-well plates to generate a confluent monolayer. The scratch was generated on the confluent monolayer by scraping with a 200 μl pipette tip and incubated with FBS-free medium at 37°C for 48 h. The cells in the wounded monolayer were photographed at 0, 24, and 48 h after scratching, and cell migration was assessed by quantifying the gap sizes at which transfected cells migrated from the edge of the scratch toward the center.

Cell invasion assays were carried out using Transwell chambers (Corning, NY, United States). A total of 100 μl serum-free medium containing 5 × 10^4^ cells was added to the upper chamber with a precoated thin layer of a basement membrane matrix, and 500 μl 10% FBS culture medium was added to the lower chamber. After incubation for 24 h, non-invaded cells on the top of the Transwell were removed, and cells that invaded through the filter were stained with crystal violet staining solution (0.1% w/v, Beyotime Biotechnology, China) and photographed under a microscope. The migrated cells in all images were counted by using ImageJ 1.48v software (National Institutes of Health, United States). All assays were repeated independently three times.

### Cell Viability Assay

Cell viability was assessed by a Cell Counting Kit-8 (CCK-8; HY-K0301, MedChem Express, United States) according to the manufacturer’s instructions. Briefly, transfected cells were seeded in triplicate in 96-well plates at a density of 5,000 cells per well and treated with different concentrations of gemcitabine for 48 h. Then 10 μl of the CCK-8 solution was added to each well of the plate and incubated at 37°C for 1 h. Optical densities (ODs) were then measured at 450 nm using a Multiskan^TM^ FC Microplate Photometer (Thermo Scientific, United States). The experiments were performed three times for quantification. The half maximal inhibitory concentration (IC50) values were obtained using the IBM SPSS Statistics 16.0 software (SPSS Inc., Chicago, IL, United States).

### Cell Cycle and Apoptosis Analyses

The fractions of quiescent and proliferating cells fractions can be identified by assessing the expression of proliferation-associated proteins such as Ki-67, which is rarely detected in G0-phase cells, but highly expressed in proliferating cells (G1, S, G2/M phases) (Kim and Sederstrom, 2015). The transfected cells were collected after trypsinization and washed twice with PBS. Then, single-cell resuspensions (1 × 10^6^ cells/ml) were fixed in pre-cooled 70% ethanol and stored at −20°C for 12 h, followed by staining with pre-diluted Ki-67-FITC or isotype control (BD Pharmingen, United States) in the dark for 30 min at room temperature (Kim and Sederstrom, 2015). Subsequently, the cells were washed with PBS and incubated with 500 μl PI/RNase staining solution (PBS supplemented with 50 μg/ml propidium iodide, 100 μg/ml RNase, and 2 mM MgCl_2_) for 30 min at 4°C. Cells were analyzed by flow cytometry and 20,000 events were collected per sample using a CytoFLEX flow cytometer (Beckman Coulter).

For cell apoptosis analysis, the transfected cells were seeded in 6-well plates to reach a confluence of 50–60%. Gemcitabine was then added at the concentration of IC50. After incubation for 48 h, cells were collected and stained by using an Annexin V/PI apoptosis kit (MultiSciences Co., Ltd.). Apoptosis was analyzed with the CytoFLEX flow cytometer (Beckman Coulter) and CytExpert software according to the manufacturer’s recommended protocol. The results are presented as viable cells (Annexin V−/PI−), early apoptotic cells (Annexin V+/PI−), late apoptotic/necrotic cells (Annexin V+/PI+) and late necrotic/dead cells (Annexin V−/PI+). All assays were repeated independently three times.

### RNA Isolation, Reverse Transcription, and RT-qPCR

Total RNA was isolated from pancreatic cancer cell lines using a TaKaRa MiniBEST Universal RNA Extraction Kit (Cat. #9767, TaKaRa, Dalian, China) and reverse transcribed using PrimeScript^TM^ RT Master Mix (Perfect Real Time) (Cat. #RR036Q, TaKaRa, Dalian, China). The obtained cDNA was subjected to real-time PCR using SYBR^®^ Premix Ex Taq TM II (Cat. #RR820A, TaKaRa, Dalian, China) according to the manufacturer’s recommended protocol. Specific primer sequences for CIRBP and GAPDH are shown in Supplementary Table 1. The cycle threshold (CT) value for each gene was normalized to the CT value of GAPDH, and the relative fold change in expression with respect to a reference sample was calculated by the 2^–ΔΔ*CT*^ method using ABI7500 software v2.0.6 (Applied Biosystems).

### Western Blot Analysis

Western blot analysis was performed as described in a previous study (Zhao et al., 2017). Briefly, total proteins were extracted by cold radioimmunoprecipitation assay lysis buffer (cat. no. P0013B; Beyotime Biotechnology, China) containing protease and phosphatase inhibitor cocktails. The protein concentration was measured with a bicinchoninic acid (BCA) Protein Assay Kit (cat. no. P0012; Beyotime Biotechnology, China). Protein samples (20 μg) were equally loaded and separated by 10% sodium dodecyl sulfate-polyacrylamide gel electrophoresis (SDS-PAGE). After electrophoretic separation, the samples were transferred onto polyvinylidene difluoride membranes (cat. no. IPVH00010; Millipore; Merck KGaA) and then blocked in 5% non-fat milk. The membranes were incubated with primary antibodies specific for CIRBP, DYRK1B, p27, p21, cyclin D1, cyclin A2, phosphorylated-p38, p38, phosphorylated-ERK1/2, ERK1/2, phosphorylated-mTOR, mTOR, phosphorylated-AKT, AKT, phosphorylated- p70S6K, p70S6K, bax, bcl-2, cleaved Caspase-3, β-actin and GAPDH for 12 h at 4°C and were then incubated with a horseradish peroxidase (HRP)–conjugated secondary rabbit or mouse antibody (Cell Signaling Technology, United States) incubated for 1 h at room temperature. The details of each antibody used in this study are listed in Supplementary Table 2. Protein bands were visualized using BioSci^TM^ SuperLimit ECL Chemiluminescent Substrate (Dakewei Biotechnology Co., Ltd., Shanghai, China) and photographed using a ChemiDoc Imaging System (Bio-Rad Laboratories, Inc.). Densitometric analysis was performed using Quantity One 4⋅62 (Bio-Rad, Hercules, CA, United States).

### Xenografted Tumor Models

BALB/c nude mice (6–8 weeks old) were purchased from Beijing Vital River Laboratory Animal Technology Co. (China) and randomly divided into different groups (*n* = 5 mice/group). Equal numbers (2 × 10^6^ cells) of PANC-1/Vector, PANC-1/CIRBP, PANC-1/shRNA-Vector, PANC-1/CIRBP-sh#1, and PANC-1/CIRBP-sh#2 cells were subcutaneously injected into BALB/c nude mice. The nude mice were kept in the specific pathogen-free (SPF) animal facility for 40 days. To identify the role of CIRBP in PDAC chemosensitivity *in vivo*, cells transfected with shRNA-vector or CIRBP-sh#2 lentivirus were subcutaneously injected into the right flank of BALB/c nude mice (PANC-1; 2 × 10^6^ cells). The experimental nude mice were randomly separated into four groups as follows (*n* = 5 mice/group): (1) shRNA-vector + PBS; (2) CIRBP-sh#2 + PBS; (3) shRNA-vector + Gem; and (4) CIRBP-sh#2 + Gem. When the diameter of the tumors in the shRNA-vector + PBS group reached 5 mm, gemcitabine (80 mg/kg) or PBS was administered intraperitoneally twice a week for 5 weeks. The tumor volume was calculated with the following formula: *V* = (*L* × *W*^2^)/2 (*L*, the longest tumor axis; *W*, the shortest tumor axis). At the end of the experiments, all nude mice were sacrificed, and tumors were excised and processed for histological analysis. The procedures for all animal experiments were reviewed and approved by the Ethics Committee for Animal Studies at the Sixth Affiliated Hospital of Sun Yat-sen University.

### Correlation Analysis

To explore the potential interaction factors of CIRBP, we downloaded and analyzed the gene expression profiling data regarding pancreatic cancer from the Genotype-Tissue Expression (GTEx) database (GTEx Consortium., 2015), Cancer Cell Line Encyclopedia (CCLE) databases (Ghandi et al., 2019) and Cancer Genome Atlas (TCGA) database (Weinstein et al., 2013). TCGA and GTEx datasets include RNA-Seq and reversed-phase protein array (RPPA) data from tens of thousands of tumor and normal tissues, while CCLE datasets contain a variety of RNA-Seq and RPPA protein data for cancer cell lines. Pearson’s rank correlation analysis was used to assess the relationship between CIRBP and DYRK1B expression by using R software (version 3.6.0, R Foundation for Statistical Computing, Vienna, Austria).

### Statistical Analysis

All *in vitro* experiments were performed with triplicate samples and at least three times biological replicates. Data are presented as the mean ± standard deviation (SD) values. Comparisons between two groups were analyzed by unpaired two-tailed Student’s *t*-test. One-way analysis of variance (ANOVA) with the least significant difference tests was used to compare differences between multiple groups. Survival curves were plotted by using the Kaplan–Meier method and compared using the log-rank test. Correlations between the levels of CIRBP expression and clinicopathological features were analyzed using the chi-square test or Fisher’s exact test. Factors determined to be significant for overall survival using univariate Cox regression analysis were introduced into a multivariate Cox regression model to determine independent prognostic factors. Data were graphically presented using GraphPad Prism software (version 5.0, San Diego, CA, United States) and R software (version 3.6.0, R Foundation for Statistical Computing, Vienna, Austria). Statistical analysis was performed using SPSS 16.0 software (SPSS Inc., Chicago, IL, United States). *P*-values of less than 0.05 were considered to indicate statistical significance.

## Results

### High Expression of CIRBP in Tumor Tissues Is Correlated With PADC Progression and Poor Prognosis

**FIGURE 1:**
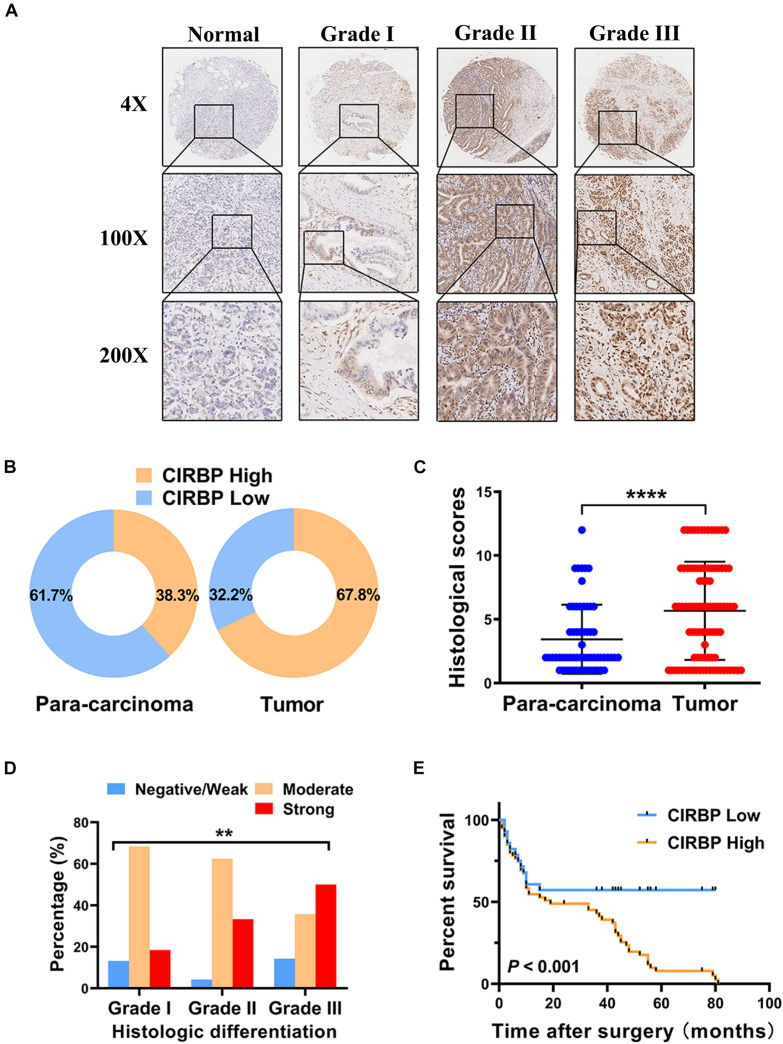
Increased expression of CIRBP is correlated with poor prognosis of pancreatic cancer patients. **(A)** Representative images of CIRBP staining in pancreatic cancer tissue microarrays with specimens at different differentiation grades or normal adjacent tissues. **(B)** CIRBP expression in human pancreatic cancer (right, *n* = 90) and corresponding para-carcinoma tissues (left, *n* = 60). The patients were divided into CIRBP-high group (histological score ≥ 3) and CIRBP-low group (histological score < 3) by histological score (staining intensity × positive percentages) according to the Fromowitz standard. **(C)** The histological scores for CIRBP staining in pancreatic cancer and para-carcinoma tissues. **(D)** The association between the expression of CIRBP and histologic differentiation grades in pancreatic cancer patients. **(E)** High CIRBP expression was associated with poor overall survival. Data are presented as means ± SD. **P < 0.01, ****P < 0.0001, unpaired student’s *t*-test.

As CIRBP exhibits a strong association with tumorigenesis in several types of cancers, we evaluated the expression levels of CIRBP in pancreatic cancer tissue microarrays containing 90 specimens of primary pancreatic cancer and 60 specimens of normal adjacent pancreatic tissue. The expression of CIRBP in PDAC specimens was much higher than that in normal adjacent pancreatic tissue specimens (Figures 1A–C). Tumors with poor histologic differentiation had a higher incidence of high CIRBP expression (total, *P* = 0.003) (Figure 1D). Moreover, Kaplan–Meier survival analysis showed that high CIRBP staining was strongly associated with poor OS (*P* < 0.001, Figure 1E), with a median survival time of 10 months in the high CIRBP staining group compared to 38 months in the low CIRBP staining group. Clinicopathological analysis showed that high CIRBP expression was associated with large tumor size and worse histologic differentiation (*P* < 0.05) (Table 1). Univariate and multivariate Cox regression analyses indicated that age, sex, tumor size and American Joint Committee on Cancer (AJCC) stage showed no prognostic significance, but lymph node metastasis (N1, *P* < 0.001), histologic differentiation (Grade III, *P* < 0.001) and CIRBP expression (High, *P* = 0.047) were independent prognostic factors for the overall survival of PDAC patients (Table 2). Furthermore, the protein and mRNA levels of CIRBP were increased in several PDAC cell lines, compared with normal human pancreatic HPDE6-C7 cells (Supplementary Figure 1). Together, these data suggest that the upregulated expression of CIRBP is associated with poor prognosis in PDAC patients.

**TABLE 1:** Associations of CIRBP expression with clinicopathological factors in pancreatic cancer patients.

Characteristics	Expression level of CIRBP			
	Low (*n* = 29)	High (*n* = 61)	χ2	*P*
Age (years)			0.163	0.687
<60	12 (41.4%)	28 (45.9%)		
≥60	17 (58.6%)	33 (54.1%)		
Gender			0.105	0.745
Female	11 (37.9%)	21 (34.4%)		
Male	18 (62.1%)	40 (65.6%)		
Tumor size (cm)			5.508	0.019
<3	16 (55.2%)	18 (29.5%)		
≥3	13 (44.8%)	43 (70.5%)		
Lymph node metastasis			0.009	0.924
N0	16 (55.2%)	33 (54.1%)		
N1	13 (44.8%)	28 (45.9%)		
AJCC stage			0.323	0.570
I	11 (37.9%)	27 (44.3%)		
II	18 (62.1%)	34 (55.7%)		
Histologic differentiation			11.837	0.003
Grade I	7 (24.1%)	5 (8.2%)		
Grade II	20 (69.0%)	32 (52.5%)		
Grade III	2 (6.9%)	24 (39.3%)		
Survival			19.727	<0.001
Live	16 (55.2%)	7 (11.5%)		
Dead	13 (44.8%)	54 (88.5%)		

*Data were presented as n of rate (%) and analyzed using Chi-square test or Fisher’s exact test. A total of 90 patients from pancreatic cancer tissue microarrays were divided into CIRBP-high group (histological score ≥ 3) and CIRBP-low group (histological score < 3) by histological score (staining intensity × positive percentages) according to the Fromowitz standard. Statistical significance was considered as P < 0.05. AJCC, The American Joint Committee on Cancer.*

**TABLE 2:** Univariate and multivariate Cox regression analysis of 90 pancreatic cancer patients.

Characteristics	Univariate Cox		Multivariate Cox	
	HR (95%CI)	*P*	HR (95%CI)	*P*
Age (years)				
<60	1			
≥60	1.185 (0.731–1.919)	0.491		
Gender				
Female	1			
Male	1.163 (0.701–1.930)	0.558		
Tumor size (cm)				
<3	1			
≥3	1.309 (0.792–2.161)	0.293		
Lymph node metastasis				
N0	1		1	
N1	1.764 (1.083–2.872)	0.023	2.377 (1.417–3.986)	<0.001
AJCC stage				
I	1		1	
II	1.923 (1.159–3.191)	0.011	1.392 (0.549–3.527)	0.486
Histologic differentiation				
Grade I	1		1	
Grade II	1.712 (0.721–4.068)	0.223	1.575 (0.660–3.760)	0.306
Grade III	4.889 (1.965–12.162)	<0.001	4.934 (1.893–12.857)	<0.001
CIRBP expression				
Low	1		1	
High	2.764 (1.495–5.110)	<0.001	1.948 (1.010–3.755)	0.047

*A total of 90 patients from pancreatic cancer tissue microarrays were divided into CIRBP-high group (histological score ≥ 3) and CIRBP-low group (histological score < 3) by histological score (staining intensity × positive percentages) according to the Fromowitz standard. Factors determined to be significant (P < 0.05) for overall survival using univariate Cox regression analysis were introduced into a multivariate Cox regression model. HR, hazard ratio; CI, confidence interval; AJCC, The American Joint Committee on Cancer.*

### CIRBP Knockdown Inhibits PDAC Proliferation, Invasion, and the Cell Cycle *in vitro*

**FIGURE 2:**
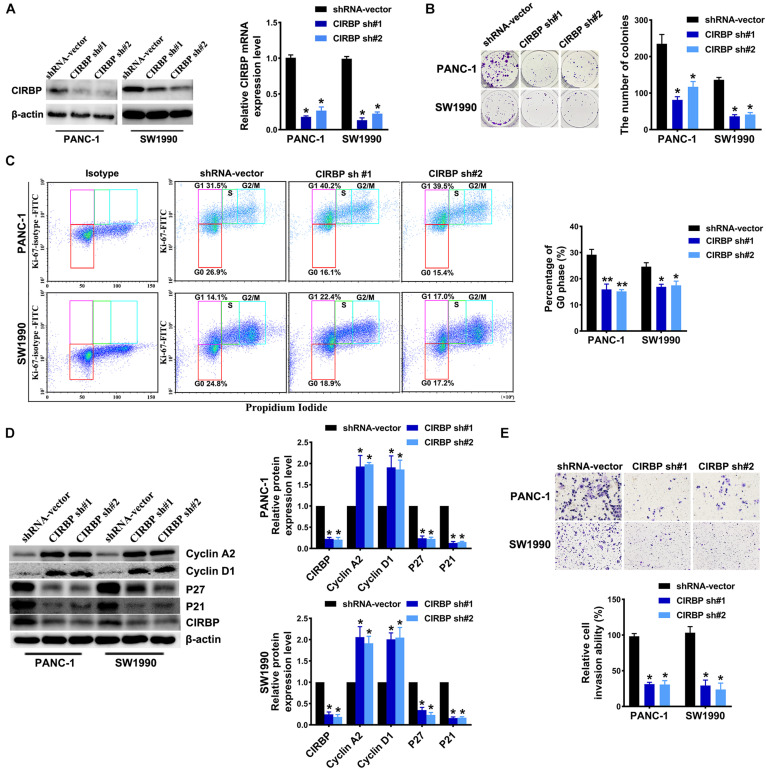
CIRBP knockdown inhibits the invasion and cell cycle of pancreatic cancer cells *in vitro*. **(A)** The interference efficiency of the lentiviral human CIRBP-targeting short hairpin RNA (shRNA) was validated by Western blot and RT-qPCR analysis. **(B)** The effects of CIRBP knockdown on the colony formation of pancreatic cancer cells. **(C)** Cell cycle of PANC-1 and SW1990 cells with CIRBP knockdown was analyzed by Ki-67 and propidium iodide stainings. Ki-67 antigen is rarely detected in G0 phase, but highly expressed in the proliferating cells (G1, S, G2, and M phases). G0 (red, lower boxes) and G1 (purple, upper left boxes) cells exhibit 2N DNA, S phase cells (green) exhibit 2N-4N DNA and G2/M cells (blue) exhibit 4N DNA. The experiments were performed on the same day and the data were analyzed with the same gating. The isotype was used as negative control. **(D)** Western blot analysis of cyclin D1, cyclin A2, p27 and p21 in CIRBP knockdown PANC-1 and SW1990 cells. β-actin was used as a loading control. **(E)** The invasion of PANC-1 and SW1990 cells with CIRBP knockdown were determined by transwell assays. Data are presented as means ± SD. *P < 0.05, **P < 0.01, unpaired student’s *t*-test.

To investigate the effects of CIRBP expression on PDAC progression, we performed CIRBP knockdown experiments in PANC-1 (PANC-1 CIRBP-sh1#/2#) and SW1990 (SW1990-CIRBP-sh1#/2#) cell lines as well as two control cell lines (PANC-1/SW1990 shRNA-vector). The protein and mRNA expression levels of CIRBP in CIRBP knockdown cell lines were significantly reduced compared to controls (Figure 2A). As shown in Figure 2B, knockdown of CIRBP in PANC-1 and SW1990 cell lines formed fewer colonies than that of controls (*P* < 0.05). The cell cycle assays showed that CIRBP knockdown led to a significant reduction in the proportions of the G0 phase population and promoted cells to re-enter the cell cycle (all *P* < 0.05, Figure 2C). The western blot assay showed that the expression levels of p27 and p21 were decreased, but cyclin D1 and cyclin A2 were increased when CIRBP expression was reduced in PDAC cells (all *P* < 0.05, Figure 2D). Moreover, wound healing and Transwell assays showed that CIRBP knockdown significantly attenuated cell migration and invasion (all *P* < 0.05, Figure 2E and Supplementary Figure 2A). Together, these data show that CIRBP knockdown reduces the malignancy of pancreatic cancer cells.

### Overexpression of CIRBP Promotes Proliferation, Invasion, and Cell Cycle Progression in PDAC Cells *in vitro*

**FIGURE 3:**
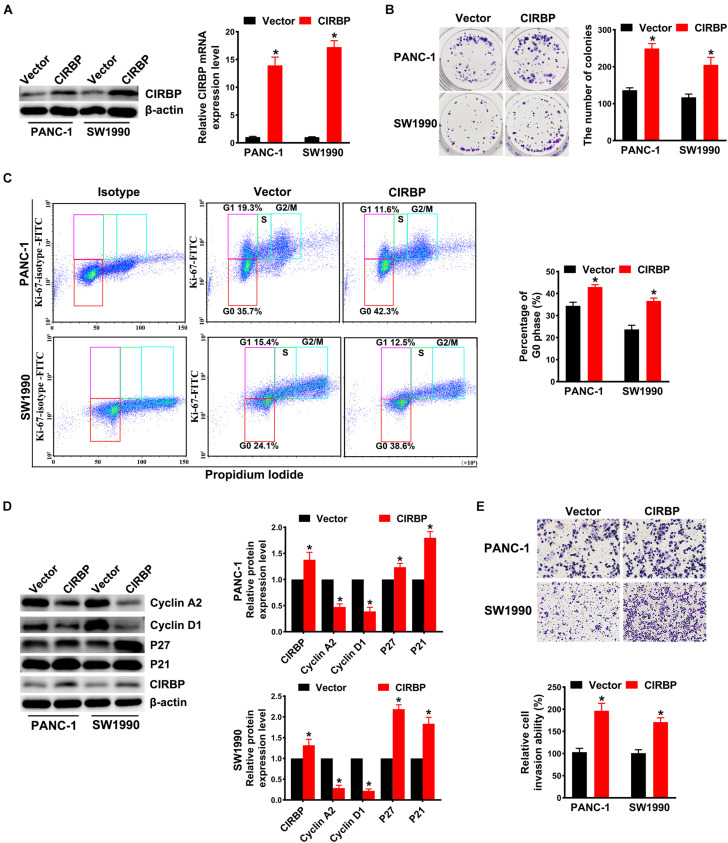
Overexpression of CIRBP promotes the invasion and cell cycle of pancreatic cancer cells *in vitro*. **(A)** The interference efficiency of the lentiviral carrying CIRBP was validated by Western blot and RT-qPCR analysis. **(B)** The effects of CIRBP overexpression on the colony formation of pancreatic cancer cells. **(C)** Cell cycle of PANC-1 and SW1990 cells with CIRBP overexpression was analyzed by Ki-67 and propidium iodide stainings. Ki-67 antigen is rarely detected in G0 phase, but highly expressed in the proliferating cells (G1, S, G2, and M phases). G0 (red, lower boxes) and G1 (purple, upper left boxes) cells exhibit 2N DNA, S phase cells (green) exhibit 2N-4N DNA and G2/M cells (blue) exhibit 4N DNA. The experiments were performed on the same day and the data were analyzed with the same gating. The isotype was used as negative control. **(D)** Western blot analysis of cyclin D1, cyclin A2, p27 and p21 in CIRBP overexpression PANC-1 and SW1990 cells. β-actin was used as a loading control. **(E)** The invasion of PANC-1 and SW1990 cells with CIRBP overexpression were determined by transwell assays. Data are presented as means ± SD. *P < 0.05, unpaired student’s *t*-test.

In addition, we generated PANC-1 and SW1990 cell lines stably overexpressing CIRBP (PANC-1/SW1990 CIRBP) as well as two control cell lines (PANC-1/SW1990 Vector). The protein and mRNA levels of CIRBP in CIRBP-overexpressing cell lines were significantly increased compared to those in control cell lines (Figure 3A). Overexpression of CIRBP in PANC-1 and SW1990 cells significantly increased the number of colonies (*P* < 0.05, Figure 3B). In addition, the overexpression of CIRBP in PDAC cells significantly increased the proportion of cells in G0 phase, accompanied by increased expression of p27 and p21 and reduced expression of cyclin D1 and cyclin A2 (Figures 3C,D). Moreover, wound-healing and Transwell assays suggested that CIRBP overexpression increased the migration and invasion capabilities of PDAC cells, compared with control cells (all *P* < 0.05, Figure 3E and Supplementary Figure 2B). These data show that CIRBP overexpression facilitates the tumourigenesis of pancreatic cancer cells.

### CIRBP Knockdown in Pancreatic Cancer Cells Inhibits Tumor Growth, While CIRBP Overexpression Promotes Tumor Growth *in vivo*

**FIGURE 4:**
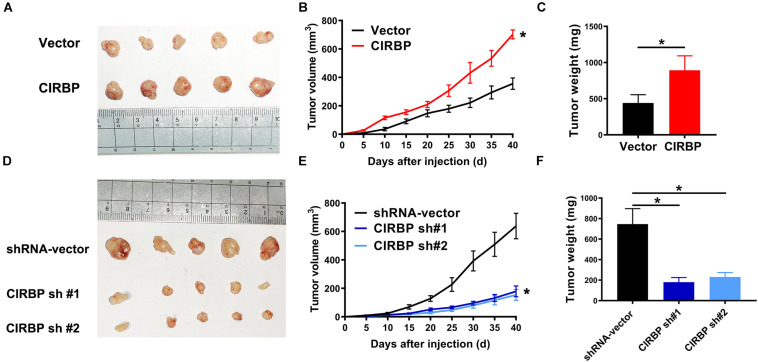
The lower levels of CIRBP expression in pancreatic cancer cells correlate with slower tumor growth *in vivo*. **(A,D)** Tumors from CIRBP overexpression **(A)** or knockdown **(D)** PANC-1 cells and their controls (*n* = 5/groups). **(B,E)** Tumor growth curves. The sizes of tumors were measured every 5 days after tumor cell inoculation. *P < 0.05, repeated measures analyses of variance with Bonferroni correction. **(C,F)** Tumor weight of each group was measured at the end of the experiments. Data are presented as means ± SD. *P < 0.05, unpaired student’s *t*-test.

Our *in vitro* studies show that increased CIRBP expression in pancreatic cancer cells facilitates their proliferation. We then tested whether the levels of CIRBP in pancreatic cancer cells will influences *in vivo* tumor growth. Tumors derived from PANC-1 cells with CIRBP overexpression exhibited a higher growth rate and larger volume than those derived from control cells (Figures 4A,B). At the end point of the experiments, the tumor weight in the CIRBP overexpression groups was higher than that in the control group (*P* < 0.05, Figure 4C). Conversely, CIRBP knockdown in PANC-1 cells suppressed tumor growth *in vivo*, resulting in lower tumor weights than those of control tumors (*P* < 0.05, Figures 4D–F). These data show that the expression levels of CIRBP in pancreatic cancer cells positively correlates with their tumor growth rate.

### CIRBP Knockdown in Pancreatic Cancer Cells Enhances Their Sensitivity to Gemcitabine Treatments

**FIGURE 5:**
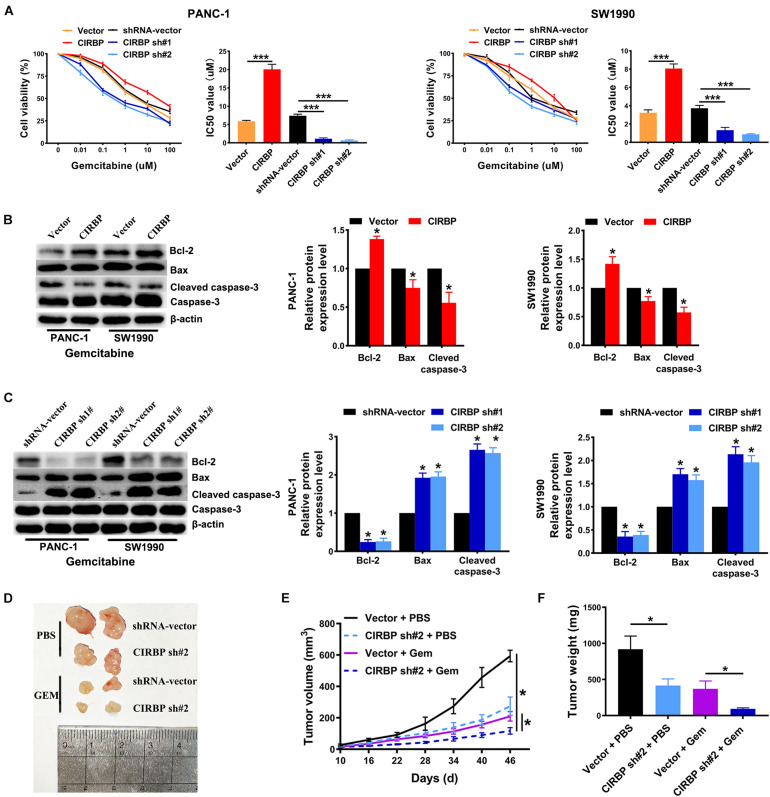
CIRBP knockdown in pancreatic cancer cells enhances its sensitivity to Gemcitabine treatments. **(A)** The IC50 of gemcitabine in PANC-1 **(left)** and SW1990 **(right)** cells were determined by CCK8 assays. The mean values of the IC50 were shown. **(B)** Western blot analysis of bcl-2, bax and cleaved caspase-3 expression in CIRBP overexpression PDAC cells after Gemcitabine treatment for 48 h. **(C)** Western blot analysis of bcl-2, bax and cleaved caspase-3 expression in CIRBP knockdown PDAC cells after Gemcitabine treatment for 48 h. **(D)** Representative tumors with corresponding treatments. Stably transfected CIRBP shRNA #2 or shRNA-vector PDAC cells were injected in nude mice, which were then divided into vector + PBS, CIRBP sh#2 + PBS, vector + Gem and CIRBP sh#2 + Gem groups and treated as described in the Section “Materials and Methods” (*n* = 5 per group). **(E)** Tumor growth curves. The sizes of tumors were measured every 6 days after tumor cell inoculation. *P < 0.05, repeated measures analyses of variance with Bonferroni correction. **(F)** Tumor weight of each group was measured at the end of the experiment. Data are presented as means ± SD. *P < 0.05, ***P < 0.001, unpaired student’s *t*-test.

Gemcitabine is the first line treatment for PDAC patients, but its efficacy is modest which is primarily due to drug resistance. To test whether CIRBP is involved in the resistance of PDAC to gemcitabine treatment, we used gemcitabine to treat pancreatic cancer cells with different levels of CIRBP expression. The viability of all lines of pancreatic cancer cells decreased gradually with increasing concentration of gemcitabine. Importantly, the IC50 value of gemcitabine was decreased in CIRBP knockdown PANC-1 and SW1990 cell lines but increased in CIRBP-overexpressing cells compared with control cells (*P* < 0.001, Figure 5A). Moreover, the proportion of apoptotic cells after gemcitabine treatment in the CIRBP knockdown group was higher than that in the vector control group but lower than that in the CIRBP overexpression group (*P* < 0.05, Supplementary Figure 3). Furthermore, we found that increased or decreased CIRBP expression alone did not alter the apoptosis rates of pancreatic cancer cells not treated with gemcitabine (Supplementary Figure 3). Interestingly, the protein levels of cleaved caspase-3 and bax were decreased in CIRBP-overexpressing PANC-1 and SW1990 cells, while that of bcl-2 was increased (Figure 5B). The protein levels of bcl-2 were decreased in CIRBP knockdown cells, but cleaved caspase-3 and bax were increased (Figure 5C). *In vivo*, tumor growth was significantly suppressed in the CIRBP knockdown group compared to the control group when the tumors were simultaneously treated with gemcitabine (*P* < 0.05, Figures 5D–F). Together, these data suggest that reduced CIRBP expression in pancreatic tumor cells improves their sensitivity to gemcitabine treatment.

### Elevated Levels of CIRBP in Pancreatic Cancer Cells Upregulate DYRK1B Expression

**FIGURE 6:**
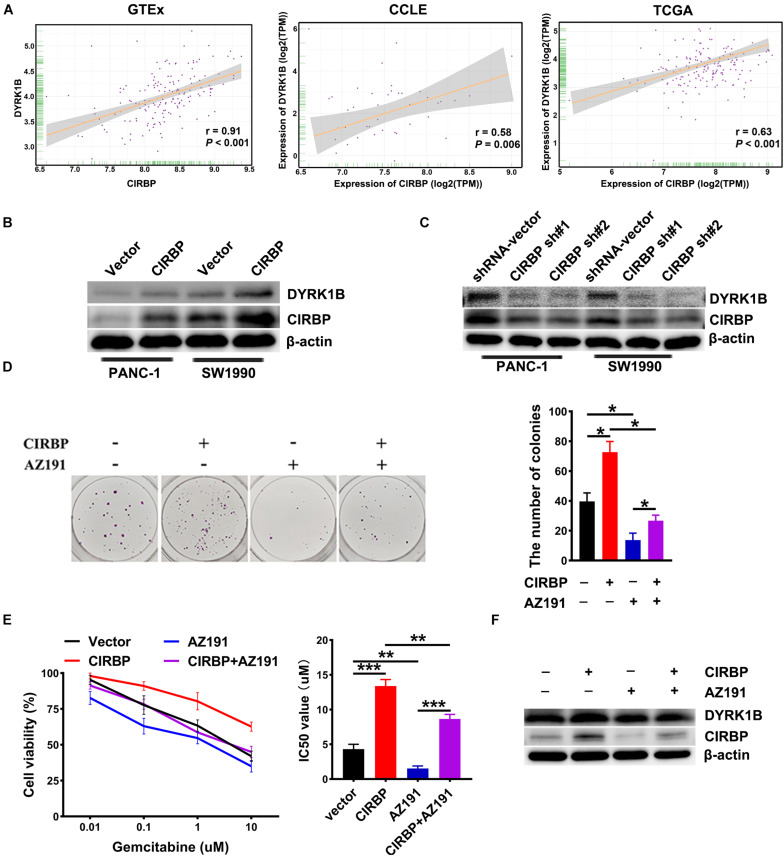
CIRBP rends pancreatic cancer cell resistance to Gemcitabine treatments by upregulating DYRK1B expression. **(A)** Three publicly available databases were used to explore the potential interaction factors of CIRBP. The Cancer Genome Atlas (TCGA) and Genotype-Tissue Expression (GTEx) datasets include RNA-Seq data for tens of thousands of tumors and normal tissues; the Cancer Cell Line Encyclopedia (CCLE) dataset provides a variety of cancer cell-line RNA-Seq data. Data were analysis using Pearson’s rank correlation analysis. **(B)** The expression of DYRK1B in CIRBP overexpressed PANC-1 and SW1990 cells was analyzed by Western blot. **(C)** The expression of DYRK1B in CIRBP knockdown PANC-1 and SW1990 cells was analyzed by Western blot. **(D)** Representative images and quantification of colonies in CIRBP overexpressed PANC-1 cells with or without AZ191 (5 μM for 48 h) treatments. AZ191 is a novel selective kinase inhibitor of DYRK1B. **(E)** AZ191 treatments enhanced the sensitivity of PACN-1 cells with CIRBP overexpression to gemcitabine treatments. **(F)** The expression of CIRBP and DYRK1B in CIRBP overexpressed PANC-1 cells with or without AZ191 treatments was analyzed by Western blot. Data are presented as means ± SD. *P < 0.05, **P < 0.01, ***P < 0.001, unpaired student’s *t*-test.

To gain more insight into the mechanism of CIRBP in regulating PDAC malignancy and chemoresistance, we analyzed three public datasets and found that the expression level of CIRBP was positively correlated with that of DYRK1B (all, *P* < 0.05, Figure 6A). Knockdown of CIRBP in PANC-1 and SW1990 cells significantly decreased but CIRBP overexpression increased the protein levels of DYRK1B (Figures 6B,C). As shown in Figure 6D, AZ191, a novel selective kinase inhibitor of DYRK1B, reversed the increased number of colonies in CIRBP-overexpressing PANC-1 cells (*P* < 0.05). AZ191 treatment also increased the cytotoxic effects of gemcitabine in CIRBP-overexpressing cells, which was relatively chemoresistant compared with vector cells (*P* < 0.05, Figure 6E). Moreover, AZ191 treatments decreased DYRK1B expression not only in PANC-1 parental cells but also in CIRBP-overexpressing PANC-1 cells (Figure 6F). Together, these data suggest that CIRBP promotes PDAC progression and chemoresistance via upregulation of DYRK1B.

### CIRBP Interacts With DYRK1B and Sustains ERK^*low*^/p38^*high*^ Activity Ratios

**FIGURE 7:**
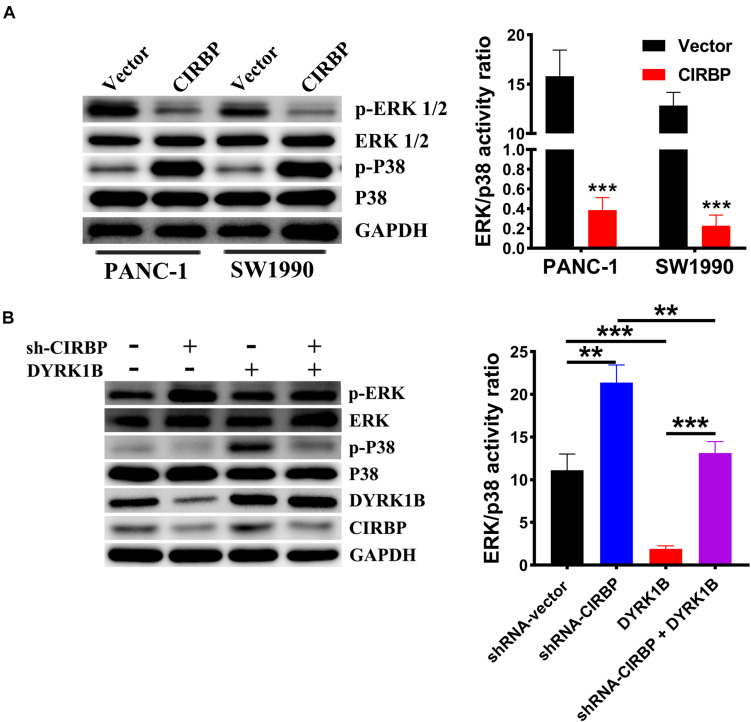
CIRBP upregulates DYRK1B and decreases the ratios of p-ERK/p-P38. **(A)** Proteins in the mitogen-activated protein kinase (MAPK) family, including phosphorylated and total ERK1/2 and p38, were analyzed by Western blot, suggesting activation of ERK1/2^*low*^ and p38^*high*^ activity ratios by CIRBP in PANC-1 and SW1990 cells. **(B)** Western blot analysis showed that DYRK1B overexpression decreased the ratio of ERK/p38 in CIRBP sh#2 (sh-CIRBP)-transfected PANC-1 cells. The ratio of ERK/p38, expressed as the value of p-ERK to p-P38, which the value of phosphorylated ERK1/2 or p38 was standardized by total ERK1/2 or p38 before, respectively. GAPDH was used as a loading control. Data are presented as means ± SD. **P < 0.01, ***P < 0.001, unpaired student’s *t*-test.

Compared with control cells, the expression levels of phosphorylated ERK were decreased, but phosphorylated p38 was increased in CIRBP-overexpressing PANC-1 and SW1990 cells, while the reverse results were shown in CIRBP-depleted PANC-1 cells (*P* < 0.05, Figures 7A,B). DYRK1B overexpression strongly activated the phosphorylation of p38. Loss of CIRBP resulted in an increased ERK/p38 activity ratio, while overexpressed DYRK1B decreased the ERK/p38 activity ratio in CIRBP-silenced PANC-1 cells (*P* < 0.05, Figure 7B). In addition, the expression levels of phosphorylated AKT and phosphorylated mTOR were increased in CIRBP-overexpressing PANC-1 and SW1990 cells compared with control cells, and the opposite results were shown in CIRBP knockdown PANC-1 and SW1990 cells. However, the level of phosphorylated p70S6K was not regulated by CIRBP (Supplementary Figure 4).

## Discussion

Pancreatic ductal adenocarcinoma patients have an extremely poor prognosis and usually endure a considerable disease burden. Over the last two decades, only a few improvements have been achieved in PDAC treatment modalities. Therefore, more insights into the mechanisms of tumorigenesis and chemoresistance in PDAC may unveil novel targets to control PDAC tumors. In this study, we demonstrated that CIRBP plays an oncogenic role in promoting the proliferation, migration, and invasion of PDAC both *in vivo* and *in vitro*. Moreover, upregulation of CIRBP in PDAC confers resistance to gemcitabine treatment. Mechanistically, CIRBP facilitates PDAC tumorigenesis and resistance to gemcitabine therapy via upregulation of DYRK1B and a reduction in the ERK/p38 activity ratio.

CIRBP has been identified as an important oncogene that plays critical roles in proliferation, invasion, and metastasis in prostate cancer, liver cancer, breast cancer and bladder cancer (Lujan et al., 2018). Our present data showed that patients with PDAC had a poor prognosis when the expression of CIRBP was upregulated, indicating the significance of CIRBP in PDAC pathogenesis. In pancreatic cancer cell lines, we demonstrated that CIRBP overexpression promoted PDAC cell proliferation and metastasis, and these effects could be reversed by CIRBP knockdown. Consistent with previous studies showing that CIRBP involved in drug metabolism and chemoresistance (Zhu et al., 2016), the levels of CIBRP expression in PDAC cells were inversely correlated with their chemosensitivity to gemcitabine treatments. In addition, our results demonstrated that CIRBP induced a higher proportion of PDAC cells to enter a quiescent state, and its deletion forced them to exit the quiescent state. Although quiescent cancer cells showed a relatively longer cycle of doubling time response to crowding microenvironmental conditions and depletion of critical nutrients or growth factors (Endo and Inoue, 2018), we also confirmed that CIRBP overexpression promoted the proliferation of PDAC cells *in vitro* by colony assays. It is possible that the quiescent cells induced by CIRBP did not modulate the *in vitro* proliferation rate of cancer cell monolayers as reported in the previous studies (Peters et al., 2003; Roesch et al., 2010). Consistent with these observations, a previous study indicated that slow-cycling cancer cells formed more and larger colonies, which showed increased self-renewal *in vitro* (Roesch et al., 2010). The characteristics of this low-density cell growth test could be interpreted as those in which disseminated tumor cells (DTCs) are exposed after extravasation in secondary colonization sites, which are usually quiescent and undetectable but have the capacity to drive long-term propagation and recurrence (Crea et al., 2015; Guereno et al., 2020). We speculated that these cells might exit quiescence and proliferate when they are cultured at low density with adequate nutrition. This possibility could also explain the increased invasion ability of CIRBP-overexpressing PDAC cells shown in the Transwell assays. These findings provide novel evidence for an oncogenic role of CIRBP in PDAC.

Previous studies suggest that CIRBP plays oncogenic roles by binding with specific mRNAs encoding cancer-associated proteins (Sakurai et al., 2015; Jian et al., 2016; Zhang et al., 2016; Lujan et al., 2018). As a novel oncogene, CIRBP has been shown to increase the expression of HIF-1α by binding to the 3′-UTR of its mRNA, which suppresses the expression of prostaglandin I synthase (PTGIS) and regulates tumor progression (Lu et al., 2018). Other proteins that interact with CIRBP and promote tumor invasion and chemoresistance remain to be explored. To better understand its molecular mechanisms in PDAC tumourigenesis, we analyzed publicly available resources, such as transcriptional profiles from the GTEx project, TCGA, and CCLE database, and found that DYRK1B is a potential interactive protein of CIRBP. It has been reported that CIRBP directly binds to DYRK1B, and inhibits its binding to p27, resulting in decreased phosphorylation and destabilization of p27 (Masuda et al., 2012). Our data showed that the levels of CIRBP were positively correlated with DYRK1B expression. CIRBP knockdown decreased the expression of DYRK1B in PDAC cells. Moreover, overexpression of CIRBP prompted colony formation of PDAC cells which was reversed by treatment with AZ191, a specific DYRK1B kinase inhibitor, treatments. These results suggest that may interact with DYRK1B in PDAC.

DYRK1B functions as a checkpoint kinase that controls the switch from a non-cycling G0 state to S phase of the cell cycle (Becker, 2018). Knockdown of DYRK1B resulted in a reduced number of quiescent cells (also termed G0-phase cells), which promoted cell cycling, reduced clonogenic capacity, increased DNA damage, and induced apoptosis in various cell lines (Becker, 2018). Our results showed that the expression of DYRK1B in PDAC cells was downregulated when CIRBP expression was suppressed, while G0-phase cells were decreased, accompanied by an increase in S and G2/M phase cells. P27 and p21 regulate the transition form G0 to G1-S phase (Besson et al., 2006). Our data showed decreased protein expression levels of p27 and p21 in CIRBP knockdown PDAC cells but increased in CIRBP overexpression cells. Quiescent cancer cells have the characteristics of a low proliferation rate, high tumourigenicity, and chemotherapy resistance and are considered to be responsible for the progression of primary tumors and metastatic relapse (Recasens and Munoz, 2019). Traditional chemotherapies, such as 5-fluorouracil (5-FU) and gemcitabine, require active cycling cells to trigger cell death (Papamichael, 1999; Jung and Lippard, 2007). Cells that are quiescent or cycling slowly are therefore less likely to be susceptible to these drugs; therefore, a potential resistance mechanism in which CIRBP interacts with DYRK1B is to regulate slow-cycling cells to evade therapeutic agents. Gemcitabine is one of the standard therapies for patients with pancreatic cancer. Our results showed that CIRBP-overexpressing PDAC cells were resistant to this ‘therapeutic’ agent and that this resistance was reversed by the DYRK1B inhibitor. These results indicate that DYRK1B mediates the resistance of PDAC cells to gemcitabine treatment upon CIRBP overexpression.

Multiple intracellular signaling pathways are involved in cancer cell quiescence and activation, including the ERK-MAPK and PI3K-AKT-mTOR pathways, in which the ERK^*MAPK*^/p38^*MAPK*^ ratio may be the key determining factor for tumor dormancy (Jo et al., 2008; Sosa et al., 2011; Gao et al., 2017). It is known that p38 can promote growth arrest by downregulating cyclin D1 and by activating the p53 to p21 and/or p16 to Rb pathways, which play important roles in cell proliferation by regulating the G1/S and G2/M checkpoints (Bulavin and Fornace, 2004). Our results indicated that the level of phosphorylated p38 was increased in CIRBP-overexpressing cells, accompanied by decreased expression of cyclin D1 and cyclin A2, while the expression pattern was reversed after CIRBP knockdown. Activation of p38α can also cooperate with reduced ERK1/2 mitogenic signaling to induce quiescence of tumor cells (Sosa et al., 2011). In the present study, we found that CIRBP decreased the ERK^*low*^/p38^*high*^ signal ratio, which might induce the quiescent cells. Moreover, we found that DYRK1B overexpression decreased the relatively high ERK/p38 activity ratio in CIRBP knockdown PDAC cells. Given that inhibiting DYRK1B in CIRBP-overexpressing PDAC cells reversed the CIRBP-induced promotion of proliferation and apoptosis in PDAC cells, these data demonstrated that CIRBP interacts with DYRK1B to promote PDAC proliferation, migration, and chemoresistance by inhibiting the ERK/p38 signaling pathways.

In summary, we obtained both clinical and experimental evidence identifying CIRBP as an important oncogene in PDAC. CIRBP overexpression promotes PDAC cell proliferation, migration, and invasion *in vitro* and tumor growth *in vivo*. Knockdown of CIRBP increases the sensitivity of PDAC tumors to gemcitabine therapy. Mechanistically, CIRBP regulates PDAC tumourigenesis and chemoresistance by interacting with DYRK1B and inhibiting ERK/p38 signaling pathways to induce cell quiescence. Thus, this study not only uncovered a novel molecular mechanism of PDAC progression but also identified the CIRBP/DYRK1B signaling pathway as a potential new molecular target for PDAC treatment.

## Data Availability Statement

The original contributions presented in the study are included in the article/Supplementary Material, further inquiries can be directed to the corresponding authors.

## Ethics Statement

The studies involving human participants were reviewed and approved by the Institutional Human Ethics Committee of the Superchip Company (Shanghai, China). The patients/participants provided their written informed consent to participate in this study. The animal study was reviewed and approved by the Ethics Committee for Animal Studies at The Sixth Affiliated Hospital of Sun Yat-sen University (IACUC-2020070101).

## Author Contributions

SJ supervised the project, designed the experiments, and interpreted the data. XC and HX performed the experiments and analyzed the data. XW and ZZ took part in analyzing the data. XC wrote the first draft of the manuscript. SJ wrote and reviewed the manuscript. All of the authors approved the final version of the manuscript.

## Conflict of Interest

The authors declare that the research was conducted in the absence of any commercial or financial relationships that could be construed as a potential conflict of interest.

## Publisher’s Note

All claims expressed in this article are solely those of the authors and do not necessarily represent those of their affiliated organizations, or those of the publisher, the editors and the reviewers. Any product that may be evaluated in this article, or claim that may be made by its manufacturer, is not guaranteed or endorsed by the publisher.

## Supplementary Material

Supplementary Figure 1 The expression levels of CIRBP in different pancreatic cancer cell lines and the immortal human pancreatic duct epithelial cell line (HPDE6-C7). **(A)** The protein expression of CIRBP were assessed by Western blot. **(B)** The mRNA expression of CIRBP were assessed by RT-qPCR. HPDE6-C7 was used as a control of pancreatic cancer cells. ^∗^*P* < 0.05, ^∗∗∗^*P* < 0.0001, one-way analysis of variance.

Supplementary Figure 2 The influence of CIRBP on migration of PDAC cells *in vitro*. **(A)** The effect of CIRBP knockdown on migration of PANC-1 **(left)** and SW1990 **(right)** cells. **(B)** The effect of CIRBP overexpression on migration of PANC-1 **(left)** and SW1990 **(right)** cells. All experiments were repeated three times. Data are presented as mean ± SD. ^∗^*P* < 0.05, compared the relative wound residual area between the CIRBP sh#1 or sh#2 group and the shRNA-vector group, unpaired student’s *t*-test.

Supplementary Figure 3 The apoptotic analysis of PDAC cells treated with gemcitabine (Gem: PANC-1, 5 μM; SW1990, 3 μM) for 48 h. **(A)** PANC-1 cells were treated with gemcitabine at 5 μM. **(B)** SW1990 cells were treated with gemcitabine at 3 μM. Data are presented as mean ± SD. ^∗^*P* < 0.05, ^∗∗∗^*P* < 0.001, unpaired student’s *t*-test.

Supplementary Figure 4 AKT/mTOR pathway expression in the CIRBP-transfected PDAC cells. **(A)** Proteins in the AKT/mTOR pathway, including phosphorylated and total AKT, mTOR and p70S6K, were analyzed by Western blot in CIRBP overexpression PDAC cell lines. **(B)** Proteins in the AKT/mTOR pathway, including phosphorylated and total AKT, mTOR and p70S6K, were analyzed by Western blot in CIRBP knockdown PDAC cell lines. β-actin was used as a loading control.
