# Supplementary figures and images for: CIRBP Knockdown Attenuates Tumourigenesis and Improves the Chemosensitivity of Pancreatic Cancer via the Downregulation of DYRK1B
Source: Front Cell Dev Biol. 2021 Aug 20;9:667551. doi: 10.3389/fcell.2021.667551 (PMC8417580; doi:10.3389/fcell.2021.667551)

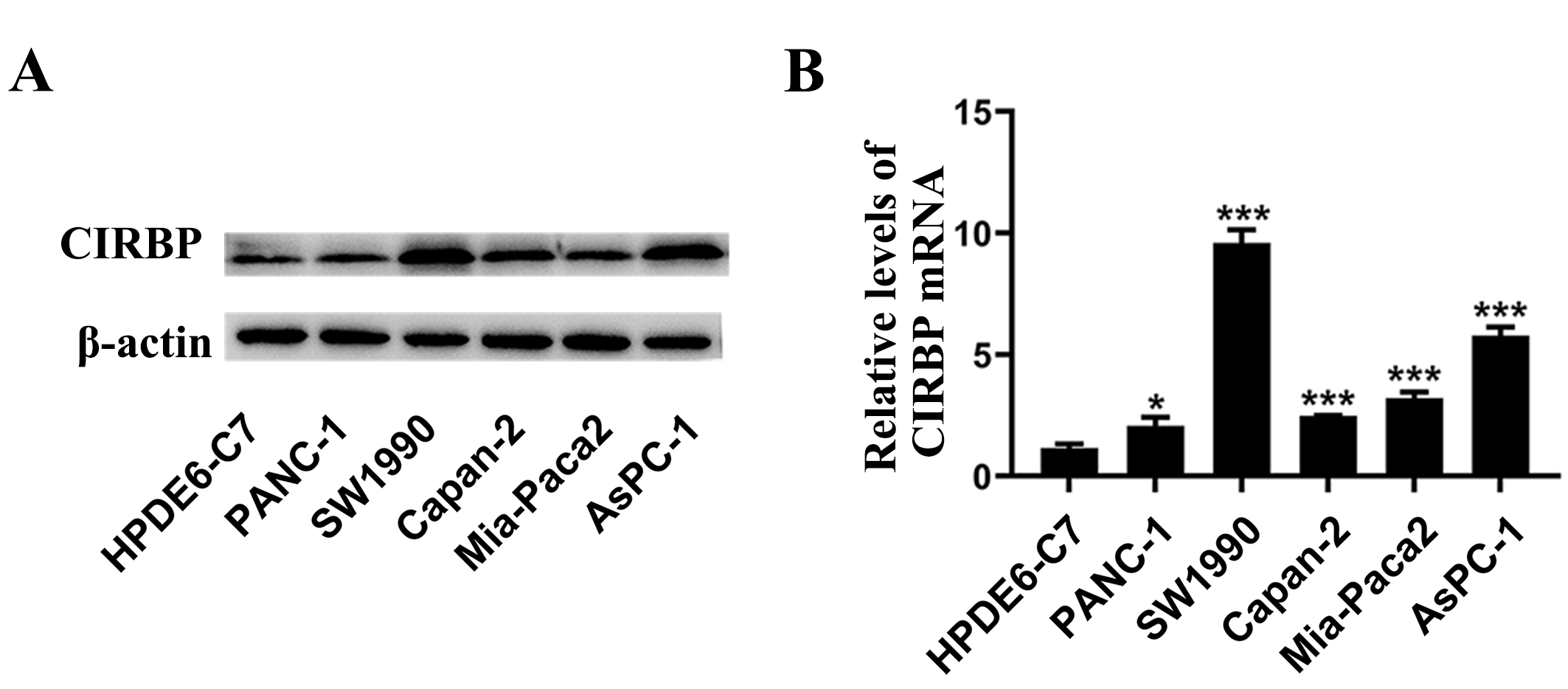

Supplement: Supplementary Figure 1 — The expression levels of CIRBP in different pancreatic cancer cell lines and the immortal human pancreatic duct epithelial cell line (HPDE6-C7). (A) The protein expression of CIRBP were assessed by Western blot. (B) The mRNA expression of CIRBP were assessed by RT-qPCR. HPDE6-C7 was used as a control of pancreatic cancer cells. ∗P < 0.05, ∗∗∗P < 0.0001, one-way analysis of variance. [file Image_1.TIF]

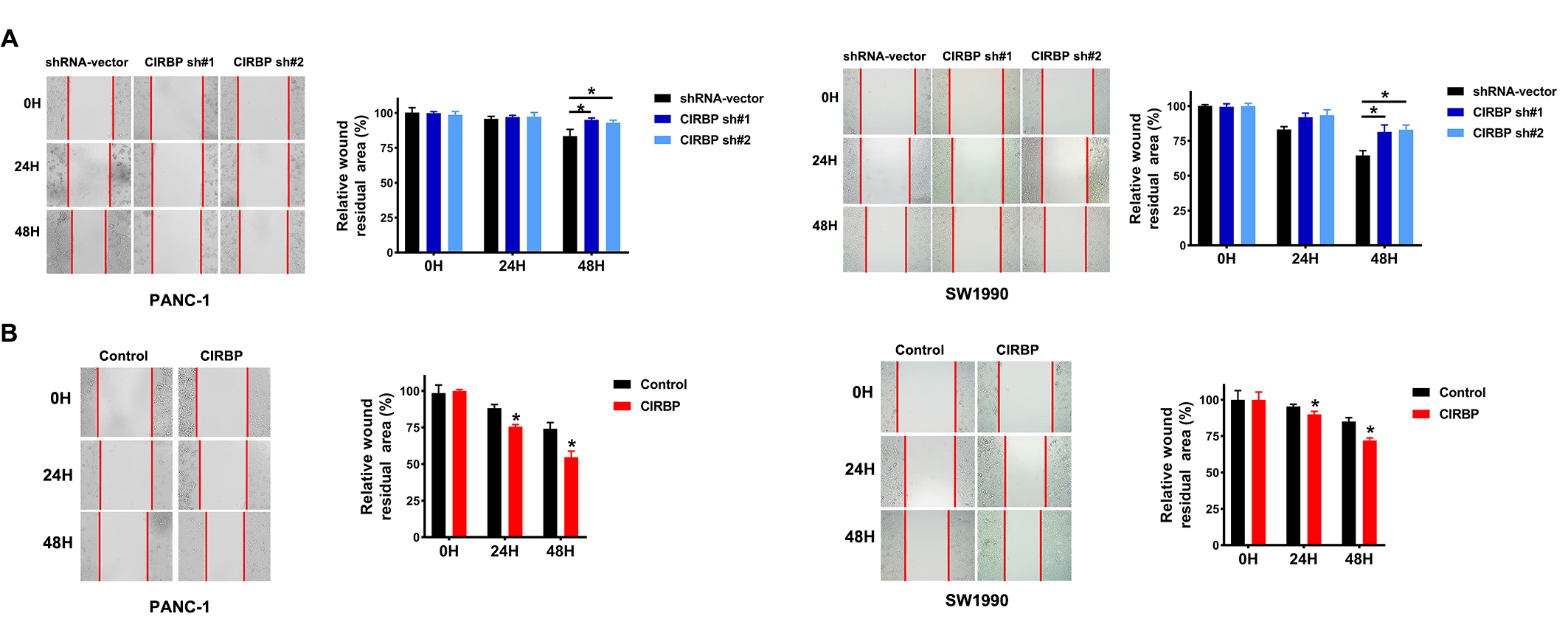

Supplement: Supplementary Figure 2 — The influence of CIRBP on migration of PDAC cells in vitro. (A) The effect of CIRBP knockdown on migration of PANC-1 (left) and SW1990 (right) cells. (B) The effect of CIRBP overexpression on migration of PANC-1 (left) and SW1990 (right) cells. All experiments were repeated three times. Data are presented as mean ± SD. ∗P < 0.05, compared the relative wound residual area between the CIRBP sh#1 or sh#2 group and the shRNA-vector group, unpaired student’s t-test. [file Image_2.TIF]

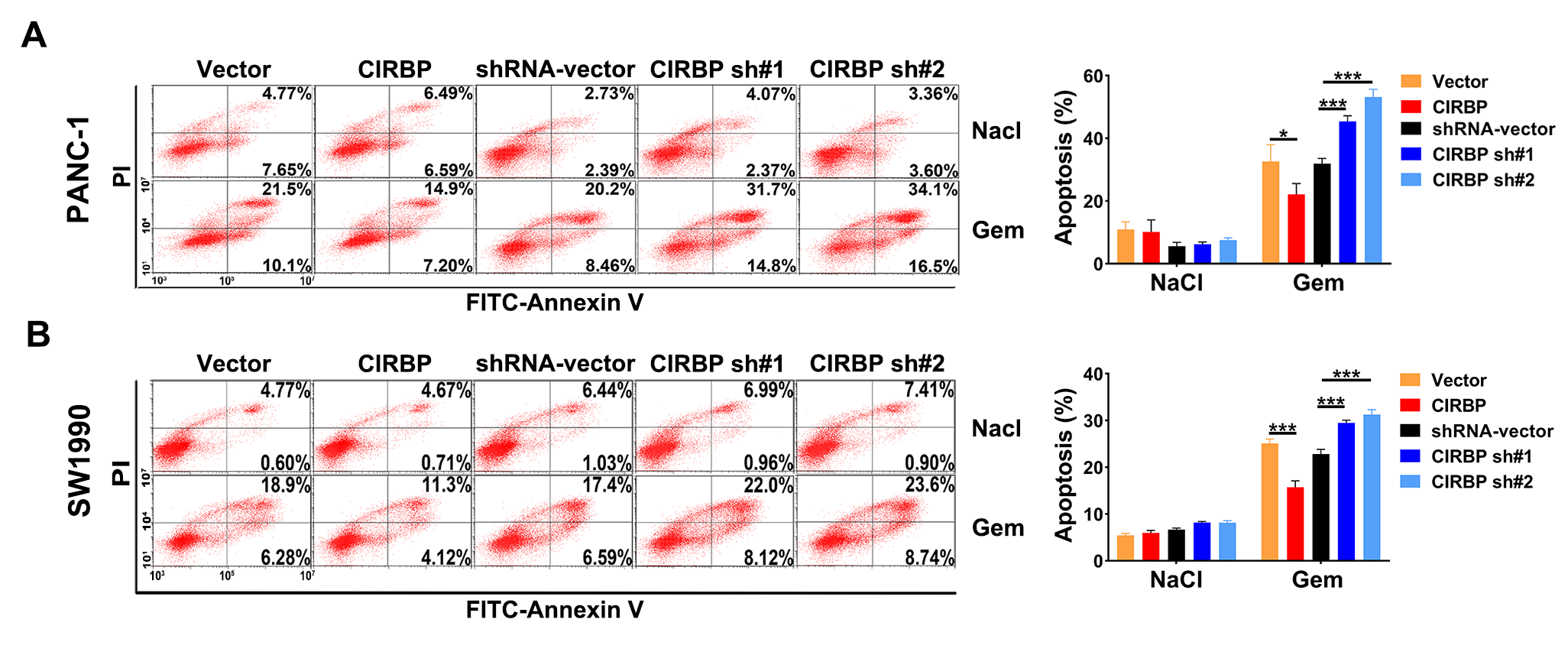

Supplement: Supplementary Figure 3 — The apoptotic analysis of PDAC cells treated with gemcitabine (Gem: PANC-1, 5 μM; SW1990, 3 μM) for 48 h. (A) PANC-1 cells were treated with gemcitabine at 5 μM. (B) SW1990 cells were treated with gemcitabine at 3 μM. Data are presented as mean ± SD. ∗P < 0.05, ∗∗∗P < 0.001, unpaired student’s t-test. [file Image_3.TIF]

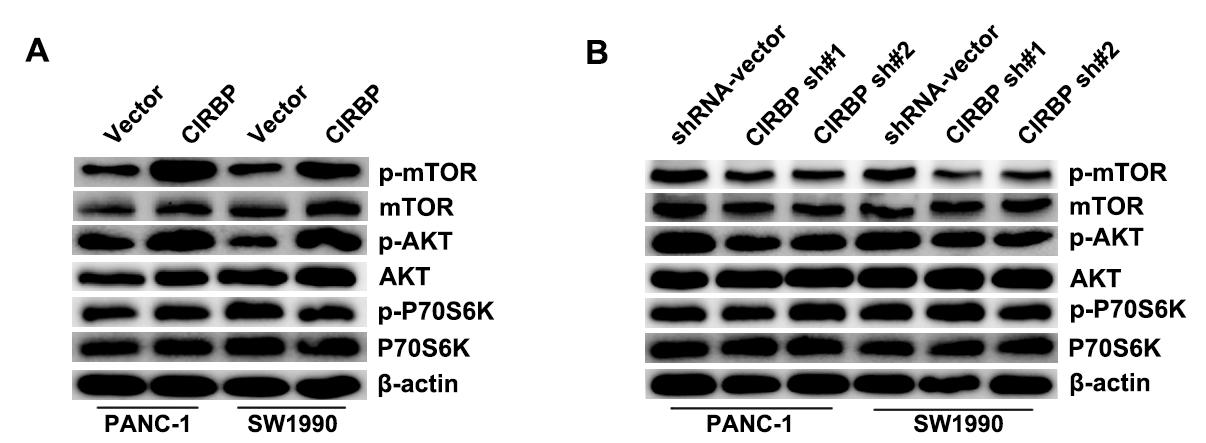

Supplement: Supplementary Figure 4 — AKT/mTOR pathway expression in the CIRBP-transfected PDAC cells. (A) Proteins in the AKT/mTOR pathway, including phosphorylated and total AKT, mTOR and p70S6K, were analyzed by Western blot in CIRBP overexpression PDAC cell lines. (B) Proteins in the AKT/mTOR pathway, including phosphorylated and total AKT, mTOR and p70S6K, were analyzed by Western blot in CIRBP knockdown PDAC cell lines. β-actin was used as a loading control. [file Image_4.TIF]
