# Supplementary material for: CIRBP Knockdown Attenuates Tumourigenesis and Improves the Chemosensitivity of Pancreatic Cancer via the Downregulation of DYRK1B
Source: Front Cell Dev Biol. 2021 Aug 20;9:667551. doi: 10.3389/fcell.2021.667551 (PMC8417580; doi:10.3389/fcell.2021.667551)
Supplement: Supplementary file 5 [file Table_1.DOCX]

**Supplementary Table S1. List of primers for qRT-PCR.**

| **Gene name** | **NCBI GeneID** | **Forward primer (5’-3’)** | **Reverse primer (5’-3’)** |
| --- | --- | --- | --- |
| CIRBP | 1153 | AGGGCTGAGTTTTGACACCAA | ACAAACCCAAATCCCCGAGAT |
| GAPDH | 2597 | GCACCGTCAAGGCTGAGAAC | GCCTTCTCCATGGTGGTGAA |

**Supplementary Table S2. List of antibodies.**

| **Antigens** | **Species antibodies raised in** | **Dilution (WB)** | **Supplier** |
| --- | --- | --- | --- |
| CIRBP | Rabbit, polyclonal | 1:1,000 | Proteintech Group, USA, Cat. # 10209-2-AP |
| DYRK1B | Rabbit, monoclonal | 1:1,000 | Cell Signaling Technology, USA, Cat. # 5672S |
| p44/42 MAPK (Erk1/2) | Rabbit, monoclonal | 1:1,000 | Cell Signaling Technology, USA, Cat. # 4695 |
| Phospho-p44/42 MAPK (Erk1/2) (Thr202/Tyr204) | Rabbit, monoclonal | 1:1,000 | Cell Signaling Technology, USA, Cat. # 4370 |
| p38 MAPK | Rabbit, monoclonal | 1:1,000 | Cell Signaling Technology, USA, Cat. #8690 |
| Phospho-p38 (Thr180/ Tyr182) | Rabbit, monoclonal | 1:1,000 | Cell Signaling Technology, USA, Cat. # 4511 |
| AKT | Rabbit, monoclonal | 1:1,000 | Cell Signaling Technology, USA, Cat. # 2920 |
| Phospho-AKT | Rabbit, monoclonal | 1:1,000 | Cell Signaling Technology, USA, Cat. # 4060 |
| mTOR | Rabbit, monoclonal | 1:1,000 | Abcam, UK, Cat. # ab32028 |
| Phospho- mTOR | Rabbit, monoclonal | 1:1,000 | Abcam, UK, Cat. # ab109268 |
| P70S6K | Mouse, monoclonal | 1:1,00 | Santa Cruz Biotechnology, USA, Cat. # sc-8418 |
| Phospho- p70S6K | Mouse, monoclonal | 1:1,00 | Santa Cruz Biotechnology, USA, Cat. #sc-8416 |
| Bax | Rabbit, monoclonal | 1:1,000 | Cell Signaling Technology, USA, Cat. # 2772S |
| Bcl-2 | Rabbit, monoclonal | 1:1,000 | Abcam, UK, Cat. # ab32124 |
| Cleaved Caspase-3 | Rabbit, monoclonal | 1:1,000 | Cell Signaling Technology, USA, Cat. # 9664S |
| P27 | Rabbit, monoclonal | 1:1,000 | Cell Signaling Technology, USA, Cat. # 3686S |
| Cyclin D1 | Rabbit, monoclonal | 1:10,000 | Abcam, UK, Cat. # ab134175 |
| Cyclin A2 | Mouse, monoclonal | 1:200 | Santa Cruz Biotechnology, USA, Cat. # sc-271682 |
| P21 | Rabbit, monoclonal | 1:200 | Santa Cruz Biotechnology, USA, Cat. # sc-6246 |
| β-actin | Mouse, monoclonal | 1:2,000 | Boster, China, Cat. # BM0627 |
| GAPDH | Mouse, monoclonal | 1:2,000 | CW Biotechnology, China, Cat. # CW0100 |
